# Macrophage migration inhibitory factor in prodromal Alzheimer’s disease

**DOI:** 10.1002/alz.088893

**Published:** 2025-01-09

**Authors:** Qumars Behfar, Stefan Kambiz Behfar, Oezguer A. Onur

**Affiliations:** ^1^ Department of Neurology, Faculty of Medicine and University Hospital Cologne, University of Cologne, Cologne, Germany, Cologne, North‐rhine Westphalia Germany; ^2^ HES‐SO University of Applied Sciences and Arts of Western Switzerland, Geneva, Switzerland, Geneva, Geneva Switzerland; ^3^ Department of Neurology, Faculty of Medicine and University Hospital Cologne, University of Cologne, Cologne, Germany, Cologne, North‐rhine westphalia Germany

## Abstract

**Background:**

Alzheimer's disease (AD) presents a prolonged asymptomatic phase, providing a significant timeframe for potential intervention. Leveraging this opportunity necessitates the early identification of diagnostic and prognostic biomarkers to detect Alzheimer's pathology during predementia stages. This enables the identification of individuals likely to progress to Alzheimer's‐type dementia, allowing them to benefit from targeted disease‐modifying therapies. The primary and critical risk factor for AD is age. As cells undergo aging, they enter a senescent state characterised by morphological alterations and the release of immune signaling mediators linked to systemic low‐grade inflammation. Therefore, we conducted a search for senescence associated markers in serum and CSF and examined their link with disease progression, aligning with clinical disease staging.

**Method:**

We assessed the levels of the available senescence‐associated secretory phenotype (SASP) factors in serum and CSF samples of cognitively normal (CN), mild cognitive impairment (MCI) and AD patients from the Alzheimer’s Disease Neuroimaging Initiative (ADNI). First, we evaluated the SASP levels in clinical stage, then we examined the association between SASP and the classical AD imaging and CSF core biomarkers, and finally we assessed the effect of SASP on the disease progression toward AD dementia.

**Result:**

CSF measures of hepatocyte growth factor (HGF), macrophage migration inhibitory factor (MIF), matrix metalloproteinase (MMP)‐3, vascular endothelial growth factor (VEGF) and serum measures of endothelial growth factor (EGF), MMP‐1, MMP‐10 and VEGF showed significantly distinguishing levels in clinical stages. We observed significant correlation between CSF measures of MIF and phospho‐tau 181 (P‐tau181) and total tau (T‐tau) and an anti‐correlation with hippocampal volume. In our longitudinal analysis, CN and MCI subjects with increased MIF showed a higher probability of progressing in the spectrum of AD dementia and a faster cognitive decline.

**Conclusion:**

Our results supports the evidence on the role of SASP in development of AD and endorse the use of cerebrospinal MIF as a senescence‐associated prognostic indicator for AD dementia, advocating for its inclusion in the current AD biomarker scheme to encompass pathological aspects beyond amyloid and tau.

